# Look back in anger: bronchial stenosis secondary to very late recurrence of breast cancer

**DOI:** 10.31744/einstein_journal/2024AI1116

**Published:** 2024-09-20

**Authors:** Felipe Marques da Costa, Augusto Kreling Medeiros, Marcos Aurélio Fonseca Magalhães

**Affiliations:** 1 BP Beneficência Portuguesa São Paulo São Paulo SP Brazil BP Beneficência Portuguesa São Paulo, São Paulo, SP, Brazil.

A 78-year-old non-smoking female patient presented to the medical office with a history of two self-limited episodes of hemoptysis occurring 8 weeks prior. She had previously undergone treatment for ductal adenocarcinoma in the right breast 17 years ago, involving mastectomy followed by chemotherapy and radiotherapy.

**Figure 1:**
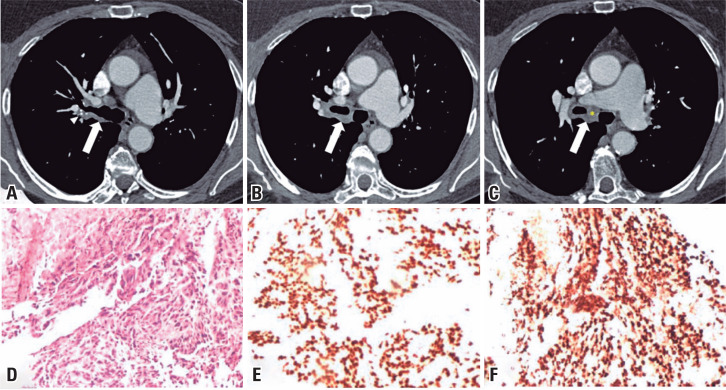
(A-C) Circumferential thickening of the right main bronchus, encompassing the origin of the upper lobe bronchus and extending to the intermediate lobe bronchus (arrows). This thickening, which promotes discrete luminal stenosis, appears to involve the airway walls, as suggested by the displacement of small cartilaginous calcifications (arrowheads). There are indications of extraparietal extension with the involvement of peribronchovascular interstitium, including the formation of hilar lymphadenopathy (asterisk); (D) Histological analysis with hematoxylin and eosin (H&E) staining presents features consistent with carcinoma; (E) Immunohistochemical photomicrography shows positivity for the GATA3 marker; (F) Immunohistochemistry demonstrates positivity for the estrogen receptor. The presence of these two markers along with the observed cellular morphology strongly indicates the presence of breast carcinoma

Physical examination revealed no abnormalities, and her SpO_2_ was 98% on room air. A CT scan exhibited circumferential thickening of the right main bronchus, encompassing the origin of the upper lobe bronchus and extending to the intermediate lobe bronchus (Figure 1 A-C). Further investigation included bronchoscopy, bronchoalveolar lavage, endobronchial biopsy, and serological tests. Immunohistochemistry results from the endobronchial biopsy revealed metastatic adenocarcinoma of the breast (Figure 1 D-F). Consequently, the patient was referred to oncology.

Bronchial stenosis, characterized by significant narrowing of the bronchial airways, can stem from various causes, including the rare occurrence of endobronchial metastases from non-pulmonary neoplasms.^(1)^ Common primary sites linked with bronchial stenosis include breast, kidney, and colorectal cancers.^(2)^ Notably, breast cancer metastasis can present as bronchial stenosis in late stages, posing a critical consideration in differential diagnosis, particularly in patients with a history of breast cancer.^(3)^ The survival rate for these patients is approximately 15 months post-diagnosis.^(2)^
